# A culturally competent approach to teaching humanities in an international medical school: potential frameworks and lessons learned

**DOI:** 10.12688/mep.18938.2

**Published:** 2022-05-11

**Authors:** Suhad Daher-Nashif, Tanya Kane

**Affiliations:** 1 The College of Medicine, QU Health, Qatar University, Doha, 2713, Qatar

**Keywords:** Culture competence, art, medical humanities, medical education, Arab region

## Abstract

**Background**: This paper describes the development of a culturally competent medical humanities course for second and third-year medical students at the ethnically diverse College of Medicine at Qatar University. First taught in 2016, the elective seminar “Medicine and the Arts” was restructured in 2017 to cultivate an appreciation of the symbiotic relationship between medicine, art, and humanities, and to foster cultural competence among the students.

**Methods**: Results and tips are based on our experiences and past reports.

**Results**: In designing a course for students immersed in an Arab-Muslim context, we encountered two challenges: the discipline’s privileging of a predominantly Western canon of arts and humanities, and the largely Euro-American-centric and unilateral framing of concepts e.g., the doctor-patient relationship, patient-centered approach, patient experiences, and meanings of health and illness. To circumvent these challenges, we followed the Purnell Model for Cultural Competence, adopted the interdisciplinary approach, and employed an intersectionality framework to build and deliver a culturally competent course exploring the nexus of arts, humanities, and medicine.

In addition to these tips on which frameworks to adopt and how to structure the course, we recommend a visual literacy workshop to help them develop the ability to recognize and understand ideas conveyed through art. Furthermore, we recommend deep conversations about artistic portrayals of medicine from different cultural contexts as tools for developing cultural awareness. Lastly, we recommend that these discussions adopt a student-centered approach, where students inform about their experiences and their own health and illness determinants, in order to develop their knowledge and practice of holism and patient centered approach, and other issues related to humanities and social sciences.

**Conclusions: **Adopting and implementing a culturally competent approach to medical education, alongside interdisciplinary and intersectionality concepts, are potential conceptual frameworks to structure a course that uses art to inform about medical humanities.

## Introduction

The benefits of introducing humanities content and concepts into medical curricula are well established, and incorporation of this field is now commonplace in the United States and Europe ^1, 2^. While some studies describe the successes and contributions of integrating humanities in medical education ^3^, several researchers and theoreticians question the long-term contributions of these programs to medical students’ skills. For example, in a literature review on “Humanities in undergraduate medical education,” Jakob and Helle examine 245 studies and conclude that evidence of the positive long-term impacts of integrating humanities into undergraduate medical education is sparse ^4^. They warn that this deficit may threaten the continued development of humanities-related activities in undergraduate medical education. While Ousager and Johannessen highlight the potential disappearance of humanities themes in medical education ^4^, Bleakley raises concerns about the counterproductive impact of these programs ^1^. In *Medical humanities and medical education: How the medical humanities can shape better doctors*, Bleakley explains that medical educators have introduced many innovations in medical curricula in an attempt to create new forms of healthcare for the coming century ^1^. These forms include professionalism, communication skills, ethics, and patient-centered care, among other themes. However, Bleakley questions the impact of these forms on the sensibilities of medical students and asserts that some of the innovations have been counterproductive. He states,

Rather than educating and widening the sensibilities of students, such innovations can produce insensibility and narrowing of focus […] Political and aesthetic dimensions are missing from medical education’s current (strongly technical) profile and medical education can have unintended negative or unproductive consequences. [ 1 p3]

Little is known about the expansion of medical humanities into non-Western contexts ^5– 7^. Those who have incorporated medical humanities in non-Western medical schools report the lack of political and aesthetic dimensions mentioned by Bleakley as well as the challenge of finding non-Western resources. This suggests that the current resources lack sensitivity to non-Western humanities. For example, Shankar and Piryani’s account of establishing a medical humanities course in Nepal cites a dearth of non-Western primary resources as a major challenge they encountered ^8^. Similarly, Hooker and Noonan criticize the narrow focus of medical humanities courses, underscore the primacy of Western cultural artifacts in their content, and problematize the medical humanities’ uncontested reliance on concepts of “difference” and “othering” ^5^. In their account of adapting a premedical ethics course delivered at an American institution in Qatar, Del Pozo and Fins mention the challenge of fostering discussion of ethics from a Western perspective while avoiding the impression that they were attempting to indoctrinate the students ^7^.

As behavioral scientists embedded within a medical school, the authors of this paper did not need to be convinced of the potential value of teaching a humanities course to medical students; we already believed in the capacity of such a course to function as a platform for fruitful discussions about the intersections between medicine, arts, health, culture, society, and politics. We were struck, however, by the limited and Western-centric nature of many of the syllabi and texts we encountered as we worked to overhaul the course.

We drew upon the experiences and insights of scholars and past studies of teaching medical humanities in Western and non-Western contexts as we grappled with similar pedagogical challenges in designing a medical humanities course in a Middle Eastern setting: the College of Medicine (CMED) at Qatar University (QU).

## Tips: Which frameworks to adopt and how to structure the course

In this section we discuss frameworks we used in building the course, and make recommendations on the structure we used to implement these frameworks.

### Guiding frameworks

To overcome others’ and our challenges, we adopted three main frameworks, namely the Purnell Model for Cultural Competence, the interdisciplinary approach to knowledge, and the intersectionality framework.

The interface between medicine and the humanities is generally considered to promote students’ cultural awareness of, and responsiveness to, specific patient populations ^9^—essential skills for physicians-in-training who will likely work as part of an interdisciplinary team and in a multicultural setting at some point in their careers. The exposure to and modeling of cultural competence starts in the classroom, where medical educators should practice what they preach. The Purnell Model (2002) suggests the following primary characteristics of cultural competence within a healthcare context ^10^:

- Developing an awareness of one’s own culture, existence, sensations, thoughts, and environment without letting these factors exert an undue influence on those from other backgrounds
- Demonstrating knowledge and understanding of the client’s culture, health-related needs, and meanings of health and illness
- Accepting and respecting cultural differences
- Not assuming that the healthcare provider’s beliefs and values are the same as the client’s
- Resisting judgmental attitudes such as “different is not as good” and navigating cultural encounters comfortably
- Adapting care to align with the client’s culture
- Developing an individualized plan of care that begins with an assessment performed through a cultural lens [ 10 p8]

Purnell’s characteristics inspired the crucial elements of the content delivered throughout the elective seminar. We anchored our course redesign in two questions: With the lens of culture focused on medical humanities, can we say that the course is taught to our specific student population in a culturally competent manner? And does the course recognize and capitalize on the rich cultural diversity that our students bring to the classroom? In our case, most members of the student body in CMED identify as ethnically Arab, while the remainder are of Asian ethnicity, predominantly Indian, Pakistani, and Bangladeshi, with many fluent in multiple languages. The majority of the students identify as Muslim.

To address these questions, we applied Purnell’s characteristics of cultural competence as we curated the materials for the course, ensuring that we used local and regional examples as our launch pads for discussions. In addition to Purnell’s Model, we adopted an interdisciplinary approach and applied intersectionality framework to examine these resources through multiple lenses and vantage points. The students were hereby introduced to the nexus between medicine, humanities (including philosophy, history, anthropology, sociology, and psychology), and visual and performing arts (fine arts, music, literature, cinema, and documentary). These intersections were then critically examined through the four lenses of the doctor-patient relationship, the patient perspective, the meaning of doctoring, and the meanings of health and illness across a range of cultures.

In pursuit of holistic understandings, we employed an interdisciplinary approach, integrating knowledge, approaches, and methods from different disciplines ^11, 12^. Medicine was conceptualized as a functional and coherent whole comprising dynamic relationships across its biological, sociocultural, political, and psychological components. We focused our inquiry on medicine and the humanities across time and place and across history and culture. Factors shaping medical practices, such as religion, class, gender, race, ethnicity, and political context, are presented in different works of art from both the East and the West. The resulting course encourages students to explore intersectionality across different socio-cultural and demographic factors that appear in works of art relevant to medical practice. The term “intersectionality” was coined in 1989 by Kimberlé Crenshaw, who challenged the notion of a universal gendered experience and argued that black women’s experiences were also shaped by race, class, and other factors. Through course discussions, the students are encouraged to consider how intersectionality between different sociodemographic factors influences the way patients, medical conditions, and practices are positioned, depicted, and manifested in both Eastern and Western works of art. Using intersectionality enabled us to incorporate most of the cultural competence course components or characteristics suggested by Purnell ^10^. We searched in vain for medical resources such as humanities texts and syllabi that did not overlook the many examples from the region where we taught the course. Hence, cognizant that we were designing the course for non-Western students immersed in a Middle Eastern context, we enriched the course content with local and regional artworks, gradually comparing them to Western and international works. This comparison deepened students’ knowledge on how society, culture, politics, and bureaucracies intersect with science, and together shape patient care, doctor-patient relationships, health perceptions, and more. Thus, the classes prompt discussions about how culture, place, and time shape the ways in which medicine is articulated in art.

### Structure of sessions

There is a reciprocal relationship between medicine and the humanities, perhaps best conceptualized as cross-fertilization. In any seminar discussion they take part in, students in the course are encouraged to interrogate this relationship through two questions. These questions mirror each other, exposing the dialectical and bidirectional relationships through which disciplines influence and are influenced by each other. For example, students are first asked to examine the intersection between the three disciplines of history (humanities), painting (fine arts), and medicine. The steering questions for this theme ask how paintings can relate the history of medical practice, how medicine has influenced painting, and what we can learn through paintings about historical approaches to the four lenses. Another example involves the intersection between psychology (humanities), cinema (arts), and medicine. The initial steering inquiry in this case asks how psychology has influenced medicine, a question that is then inverted to how medicine has influenced psychology. Table 1 illustrates the interdisciplinary and intersectionality approaches of the entire course. 

**Table 1.** The course themes.

Themes	Topics for discussion	Examples of resources
History, painting & medicine	What is history? How is the history of medicine and doctoring documented in paintings? What can paintings reveal about developments in medical practice and perceptions of the body, health, illness, and death in the East and West? What determinants impact these perceptions?	Ancient and current paintings from the East and the West. Some examples: Ibn al-Nafis, A’laa Bashir, Leonardo da Vinci, Rembrandt, and Liz Card.
Anthropology, documentary & medicine	What is anthropology? What are the connections and distinctions between medical and cultural anthropology? What has anthropology contributed to our knowledge of medicine? How is medicine explored in documentaries? How do documentaries depict medical practice, the body, the individual as human, and illness?	Documentary and ethnographic films from the East and the West: TB in Town 2 (Dr. Lianne Cremers, 2017; Rolling (Dr. Gretchen Berland, 2003) Good, B.J., 1994. How medicine constructs its objects. *Medicine,* * rationality and experience*, Cambridge University Press. Tour of the clinical skills and anatomy lab
Philosophy, literature & medicine	What is philosophy? What is literature? How does literature written by doctors reflect body, soul, and mind dialectics? How is the philosophy of medicine presented in literature?	East: al-Saadawi, N., 1958. *Memoirs of a woman doctor*. West: Sacks, O., 2007, *The Man who Mistook his Wife For a Hat*.
Sociology, music & medicine	What is sociology? What is medical sociology? What can the sociological examination of health, illness, and medical care reveal? How is music therapy represented in sociological research? What have sociological studies of music revealed about mind, body, soul, and social-mental health?	Middle Eastern music and Western music and their potential use as treatment tools with pediatric and geriatric populations. Stevens, C., 2012. *Music medicine: The science and spirit of* * healing yourself with sound*. Sounds True. Pratt, R.R. and Jones, R.W., 1987. Music and medicine: a partnership in history. In *Musik in der medizin/music in medicine* (pp. 377–388). Springer, Berlin, Heidelberg.
Psychology, cinema, television & medicine	What is psychology? How is mental health reflected in cinema? How are medical practice, the body, and illness portrayed in television?	A wide range of movie clips from around the world specifically dealing with mental health disorders. The African Doctor (2016, France); Kill Me, Heal Me (2015, South Korea); The Blue Elephant (2014, Egypt); My SO Has Got Depression (2014, Japan)

The course commences with a discussion of Damien Hirst’s local installation, “Miraculous Journey,” a series of fourteen 20-meter bronze statues depicting the gestation of a fetus inside a uterus from conception to birth. This contentious artwork is displayed outside the local women’s and children’s hospital, Sidra Medicine. Inside its walls, Sidra has dedicated spaces and exhibits artwork from different regions. Students are introduced to the medical humanities through a discussion of the issues invoked by these sculptures in their specific cultural contexts, such as the (in)appropriateness of nudity, scientific value, and public vs. private art, and more. We then begin to tackle interdisciplinary themes, as outlined in Table 1. Each thematic intersection is explored over two sessions (Sessions 1 & 2), each lasting three hours. Session 1 begins with an introduction to the relevant theoretical underpinnings of humanities and genre of art under consideration. In this part we highlight the link between humanities and medicine through art works. Furthermore, this involves a discussion of how these two disciplines (humanities and art) present medical work in combination with each other. The discussion is followed by a “show and tell” activity, wherein each student introduces their peers to an artwork produced in the same medium or genre, discussing how the piece they have chosen portrays humanities, and the four lens (the doctor-patient relationship, patient perspectives, the meaning of doctoring, and/or the meanings of health and illness). Students also consider how factors such as socio-demographic status, ethnicity, culture, gender, and politics influence the artist’s conceptualization of the topic. Session 2 elaborates on the previous session, extending the knowledge of each theme via student-led presentations of how the theme is depicted in art emanating from a Western context versus how it is manifested in an Eastern context.

The remaining sessions comprise interactive guest lectures delivered by physicians who are passionate about specific art forms, a visit to a gallery exhibit on a medical or health-related theme, and a field trip to Sidra Medicine to view art within a medical context. For example, in Spring 2021 course, Dr Amir Taj al-Sir, an accomplished Sudanese physician, novelist and poet based in Qatar was invited to talk about his experience as a doctor and novelist, and the relationship between writing and doctoring. He discussed his novel Ebola 76 and its relation to the COVID-19 pandemic and more. In previous years, students have attended visiting art installations such as the Iraqi plastic surgeon, painter and sculptor, Dr Ala Bashir internationally recognized for his portrayals of the human condition. At the end of the semester, we attend a tour of Sidra hospital to view Damien Hirst’s “Miraculous Journey” and other local, regional and international art works displayed within the hospital setting. The course culminates with an exhibition of each student’s artwork depicting the doctor-patient relationship, the patient perspective, the meaning of doctoring, or the meanings of health and illness. Each year, this art installation, displayed at the heart of the medical college, generates significant discussion among faculty and peers about the intersection of medicine, arts, and the humanities, often serving as the impetus for students to enroll in the elective seminar the following year.

## Conclusions

Medicine is art, and art represents medicine ^13, 14^. Incorporating humanities and art into the medical curriculums and programs has been reported as contributing to the development of ethics, professionalism, patient-centered approach, culture competence, holism, and more ^15^. In this paper, we have introduced and recommended the use of potential guiding frameworks to build a course on medical humanities using art as medium for teaching, and presented our concept of integrating these frameworks to foster culture competence among medical students of different nationalities in a Middle Eastern context. Informed by student feedback gathered through a post-course structured reflective questionnaire, we continue to learn new lessons to help improve the course in subsequent years. For example, we have observed that many students arriving at the College of Medicine have pursued a scientific track of academic studies with limited exposure to arts, humanities, and social sciences. This fact was reported in other reports on integrating humanities and social sciences in medical education ^6, 16, 17^. Consequently, many students lack the vernacular and knowledge to critically think humanities and/or evaluate art. To compensate for this gap, we intend to introduce a visual literacy workshop to help them develop the ability to recognize and understand ideas conveyed through art. We anticipate that the implementation of this skill-development component will facilitate more meaningful engagement, yield a deeper understanding, and foster a long-term appreciation of the reciprocal relationships across the domains of medicine, art, and the humanities. The divergent backgrounds of the two faculty members involved in the course proved to be one of its strongest assets as each brought a different lens to the material. It is beneficial to include several instructors from different cultural backgrounds in the delivery of the medical humanities course, optimizing the breadth of representation of ethic-racial content material covered. Culturally-diverse medical student populations deserve to examine contributions of art and humanities derived from marginalized and underrepresented groups to ensure inclusivity and holism. Inviting several instructors, engaging and collaborating with local physician-artists can also be a solution in contexts that lack financial resources to fund such courses. Another lesson is that conversations about artistic portrayals of medicine from different cultural contexts function as valuable tools for developing cultural awareness among medical students. The course offers the scope and space for students to participate in dialogue about social, existential, and philosophical issues such as feminism, collectivism vs. individualism, hierarchy, and patriarchy, prompting critical reflection on their own life experiences. Initial course feedback suggests that the course has fostered contemplation of these issues. For example, a second-year female said, “The course helped me to know what the patient might think of what I have just said, and how to read the patient through his facial expressions and body language.” A third year male contends, “I learned how to pay attention to details and indirect messages in art works, which I believe will help me in seeing more details when I look at real patients. It is weird to say that, but I think every patient is like an art work that I need to analyze and understand in order to give the best care.” A second year female asserts, “This is the only place where I can discuss real life issues such as women and poor people’s suffering, with no self-restriction.” The increased self-awareness and understanding of others’ perspectives ultimately translates into more culturally competent medical students. Being aware and reflective of one's personal experiences is the basis for empathy towards others’ experiences. We believe that medical humanities can plant seeds of understanding of how health and illness are firmly rooted in personal, social and political terrains, potentially yielding holistic-thinking and helping to cultivate a more patient-centered practice.

In the future, in addition to students’ feedback, we plan to use SPICES and FAIR models, as tools to evaluate and improve the course.

## Data availability

No data are associated with this article.
