# Supplementary material for: A culturally competent approach to teaching humanities in an international medical school: potential frameworks and lessons learned
Source: MedEdPublish (2016). 2022 May 11;12:6. Originally published 2022 Feb 3. [Version 2] doi: 10.12688/mep.18938.2 (PMC9370088; doi:10.12688/mep.18938.2)
Supplement: Peer review history

# Peer review: Reviewer response for version 1

**DOI:** 10.21956/mep.20295.r31706

**Published:** 2022-03-31

**Authors:** P Ravi Shankar

**Affiliations:** 1 IMU Center for Education, International Medical University, Kuala Lumpur, Malaysia

I read the article on a culturally competent approach to teaching humanities at an international medical school with interest. The authors have mentioned many of the challenges and dilemmas educators in non-western contexts have while introducing the humanities. The approach adopted by the authors has been well-described with references to the relevant literature. Their approach will be useful for authors considering medical/health humanities in non-western contexts. Art is not limited by language and may be easier to be understood across different cultures. 

 Among the different challenges in a non-western context, the first is the lesser number of available resources from a non-western context. The second that may be important in many countries is the fact that medical education is still largely delivered in English. In countries like India where there are several languages spoken finding a common language (other than English) may be difficult. Many students are educated in what is called English medium schools where all subjects are taught in English and the other languages are only learned as a subject. Many students end up being more competent in using English than their own mother tongues. Also, I believe that while there are important individual differences between cultures and regions there are also similarities brought about by the widespread use of English and the adoption of many aspects of a global culture.  

 There is a clear separation between medicine and the arts in many Asian cultures as mentioned by the authors. In Asia generally, students who do well take up Science and go for Medicine and Engineering. Those in the middle take up Commerce and go for Business related courses and Accountancy. Students who get lower grades take up the Arts. A clear dichotomy between the arts and the sciences is established early on. Arts has its own terminologies and concepts that are usually difficult to be understood by medical students. Also, medical schools may be stand-alone institutions without attached faculty of arts.    

 The suggestions by the authors in the Conclusion section appear feasible in other non-western settings. There is a restriction, however. Qatar and the institution are high-income countries with fewer problems in accessing resources. Other countries may struggle with financial resources for offering a health humanities module. Also, countries with a variety of languages may have challenges with which language is to be used during the module. Humanities may need to be presented with a lesser amount of jargon. Medical students struggle with medical jargon and concepts and adding more may make it challenging for them. Those who are interested may be offered an elective module during the course.

If evidence from practice is presented, are all the underlying source data available to ensure full reproducibility?

Yes

Is the topic of the practical tips discussed accurately in the context of the current literature

Yes

Are all factual statements correct and adequately supported by citations?

Yes

Are the conclusions drawn balanced and justified on the basis of the presented arguments?

Yes

Are arguments sufficiently supported by evidence from the published literature and/or the authors’ practice?

Yes

Reviewer Expertise:

Medical education, medical humanities, small group learning, curriculum design

I confirm that I have read this submission and believe that I have an appropriate level of expertise to confirm that it is of an acceptable scientific standard.

# Response

**Published:** 2022-04-23

**Authors:** Suhad Daher-Nashif

**Affiliations:** Qatar University, Qatar

Thank you very much for your valuable comments and reflections from your own experience in building similar courses. As you mentioned and based on our experience, the main challenge was to find nonwestern resources and artifacts for the course. Having one instructor (SDN) who have a rich knowledge related to nonwestern art works helped in reaching the artifacts. Hence, the divergent backgrounds of the two faculty members involved in the course proved to be one of its strongest assets as each brought a different lens to the material and approaches. We believe that it is beneficial to include several instructors from different cultural backgrounds in structuring and delivering medical humanities courses. This can optimize the breadth of representation of ethic-racial content material covered. Culturally-diverse medical student populations deserve to examine contributions of art and humanities derived from marginalized and underrepresented groups to ensure inclusivity and holism.  Inviting several instructors, engaging and collaborating with local physician-artists can also be a solution in contexts that lack financial resources to fund such courses. In addition, we can see the impact of the English language dominance in nonwestern medical schools, especially when it comes to the students’ interaction with non-English speaking patients/families.

 Nevertheless, in medical humanities course we can also witness the benefit of thinking and reflecting in the non-native language, as liberating from existing cognitive structures. We don’t have evidence for that yet, but we could see creativity that we could not see in other venues in the college. Furthermore, we found it easier for students to talk about sensitive or taboo issues in strange language, because it sounds more formal when compared to using the mother tongue to express the same issues.

# Peer review: Reviewer response for version 1

**DOI:** 10.21956/mep.20295.r31554

**Published:** 2022-02-17

**Authors:** Jeni Harden

**Affiliations:** 1 Usher Institute, The University of Edinburgh, Edinburgh, UK

The authors present a very interesting paper, giving tips for developing medical humanities courses. This is a challenging area of the curriculum and little has been written about how to teach medical humanities in non-Western contexts. Their tips are of relevance and value to anyone working in this field. It is particularly useful that the authors outline the frameworks that guided the development of the course. Information is also provided on the structure of the session, demonstrating how the frameworks were applied in practice. It would have been useful to hear more about the 'remaining sessions' that are mentioned very briefly. It would also be interesting to hear a little more about the students' response to the course - they are being asked to engage with a range of new disciplines and apply modes of thinking and working that may be quite out of their comfort zones. Did they see the relevance? Did they engage with intersectionality on a personal level?  A very interesting report, which raises many additional questions for us all to think about when designing such courses.

If evidence from practice is presented, are all the underlying source data available to ensure full reproducibility?

Yes

Is the topic of the practical tips discussed accurately in the context of the current literature

Yes

Are all factual statements correct and adequately supported by citations?

Yes

Are arguments sufficiently supported by evidence from the published literature and/or the authors’ practice?

Yes

Reviewer Expertise:

Social science; qualitative research

I confirm that I have read this submission and believe that I have an appropriate level of expertise to confirm that it is of an acceptable scientific standard.

# Response

**Published:** 2022-04-23

**Authors:** Suhad Daher-Nashif

**Affiliations:** Qatar University, Qatar

Thank you very much for your valuable comments. We also believe that the process, the frameworks and the structure can be a helpful example to build similar courses, especially in colleges with multicultural backgrounds and students of different nationalities.  Regarding the remaining sessions, these sessions comprise interactive guest lectures delivered by physicians who are passionate about specific art forms, a visit to a gallery exhibit on a medical or health-related theme, and a field trip to Sidra Medicine to view art within a medical context.

**For example,** in Spring 2021 course, Dr Amir Taj al-Sir, an accomplished Sudanese physician, novelist, and poet based in Qatar was invited to talk about his experience as a doctor and novelist, and the relationship between writing and doctoring. He discussed his novel Ebola 76 and its relation to the COVID-19 pandemic and more. In previous years, students have attended visiting art installations such as the Iraqi plastic surgeon, painter and sculptor, Dr Ala Bashir internationally recognized for his portrayals of the human condition. At the end of the semester, we attend a tour of Sidra hospital to view Damien Hirst’s “Miraculous Journey” and other local, regional and international art works displayed within the hospital setting. Initial course feedback suggests that the course has fostered contemplation of these issues. For example, a second-year female said, “The course helped me to know what the patient might think of what I have just said, and how to read the patient through his facial expressions and body language.” A third year male contends, “I learned how to pay attention to details and indirect messages in art works, which I believe will help me in seeing more details when I look at real patients. It is weird to say that, but I think every patient is like an art work that I need to analyze and understand in order to give the best care.” A second year female asserts, “This is the only place where I can discuss real life issues such as women and poor people’s suffering, with no self-restriction.” Students engaged with the intersectionality spontaneously, even without directing them to do so. Talking about their own experiences, and linking these experiences to their sociocultural and political determinants, created the relevance and meaning of the course’s content and themes. In the future, in addition to students’ feedback, we plan to use SPICES and FAIR models, as tools to evaluate and improve the course. We will add these examples of sessions, and students’ feedback, and evaluation tools in the revised version.
